# Children with FASD—Evolving Patterns of Developmental Problems and Intervention Costs in Ages 0 through 16 in Finland

**DOI:** 10.3390/children10050788

**Published:** 2023-04-27

**Authors:** Laura Mirjami Jolma, Mikko Koivu-Jolma, Anne Sarajuuri, Paulus Torkki, Ilona Autti-Rämö, Heli Sätilä

**Affiliations:** 1 Faculty of Medicine, University of Helsinki, Haartmaninkatu 8, P.O. Box 63, 00014 Helsinki, Finland; 2 Division of Child Neurology, Päijät-Häme Central Hospital, Keskussairaalankatu 7, 15850 Lahti, Finland; 3 Faculty of Science, University of Helsinki, Gustaf Hällströminkatu 2, P.O. Box 64, 00014 Helsinki, Finland; mikko.koivu-jolma@helsinki.fi; 4 Division of Child Neurology, Helsinki University Central Hospital, University of Helsinki, Stenbäckinkatu 11, P.O. Box 22, 00014 Helsinki, Finland

**Keywords:** fetal alcohol spectrum disorder, FASD, age-specific, cost estimation, life history

## Abstract

This is a retrospective chart review of 55 persons (mean age 11 years, range 2–28 years) diagnosed with fetal alcohol spectrum disorder (FASD) in one Finnish central hospital. The aim was to determine typical problems and interventions and estimate their costs during different periods of childhood between ages 0 and 16. During the first year, 29/38 (76.3%) were treated in the neonatal intensive care unit, 29/43 (67.4%) received physiotherapy, 15/43 (34.9%) were diagnosed with congenital malformation, 8/43 (18.6%) had heart defects. Between 1 and 6 years, 39/45 (86.7%) received occupational therapy, 25/45 (55.6%) speech therapy, and 12/45 (26.7%) were diagnosed with strabismus. Between 7 and 12 years, 25/37 (67.6%) were diagnosed with ADHD and special education was recommended for 30/37 (81.1%). Learning disorders and the need for psychiatric care increased with age. Between 13 and 16 years, 12/15 (80%) were treated in the psychiatric unit, and by this age, 8/15 (53.3%) were diagnosed with intellectual disability. Before 16 years, 44/55 (80%) were placed out of home, which caused 78.5% of the estimated cumulative mean extra costs of EUR 1,077,000 in 2022 currency. Except for psychiatric costs, health care costs were highest during early years. Charting typical patterns of problems may help in identifying children with FASD and planning follow-ups, content of assessments, and interventions.

## 1. Introduction

### 1.1. Classification of Fetal Alcohol Spectrum Disorders (FASD) and Its Subtypes

FASD is a spectrum of structural, neurobehavioral, and developmental disorders that are primarily caused by harmful effects of prenatal alcohol exposure [1], but some of the later emerging behavioral and psychiatric symptoms associated with FASD may be partially caused by interaction with the postnatal environment [2]. FASD has been divided into fetal alcohol syndrome (FAS), partial fetal alcohol syndrome (PFAS), alcohol-related neurodevelopmental disorder (ARND), and alcohol-related birth defects (ARBD) [1,3]. There is an ICD-10 code only for the dysmorphic fetal alcohol syndrome Q86.0 including FAS and PFAS, which has made diagnosing and retrospective research using registry data difficult, as ARND is the most common type of FASD [4,5]. In the ICD-11, which is in the process of being adapted, ARND is under the diagnosis 6A0Y “Other specified neurodevelopmental disorders”.

### 1.2. Diagnostic Criteria for FASD

There are multiple different diagnostic criteria for FASD used in the world with subtle differences that are beyond the scope of this study [5]. In Finland, the Institute of Medicine’s (IOM) 2005 criteria for FASD [1] have been most widely used, because Finland participated in a CIFASD study where those criteria have been validated in the Finnish population [6]. A diagnosis of FAS according to IOM criteria includes the following four components: (1) A characteristic pattern of minor facial anomalies, (2) Prenatal and/or postnatal growth deficiency, (3) Deficient brain growth, abnormal morphogenesis, or abnormal neurophysiology, and (4) Neurobehavioral impairment, meaning cognitive, or behavioral impairment. The diagnosis of PFAS is similar to that of FAS, but it does not require criterion (2) Prenatal and/or postnatal growth deficiency. In IOM’s 2016 criteria, FAS and PFAS can be diagnosed with or without documented prenatal alcohol exposure. ARND always requires documented prenatal alcohol exposure and (4) Neurobehavioral impairment.

In this study population, the IOM’s 2005 criteria were used for those diagnosed before 2017, and for those diagnosed 2017 or later, the IOM’s 2016 criteria were used. The main difference between the use of IOM 2005 and IOM 2016 criteria in Finland is that the 2016 criteria allow diagnosing FAS and PFAS even when documented maternal alcohol use during pregnancy is not available, which sometimes is the case in adopted or fostered children.

### 1.3. The Prevalence of FASD

Using school-based active-case-ascertainment studies, the prevalence of FASD was determined to be 1–5% in countries and areas with similar female alcohol consumption amounts and patterns as in Finland [4,7], but currently most remain undiagnosed. In the Finnish Care Register for Health Care, for those born in 2000–2020, the prevalence of the ICD-10 diagnosis of Q86.0 is 0.036% (424/1172208) (personal communication 5th of October 2021, Mika Gissler, Finnish Institute for Health and Welfare).

### 1.4. Health, Societal and Economic Significance of FASD

FASD has been considered the leading preventable cause of developmental disabilities in the world [3]. Many different health, developmental, and psychiatric problems have been associated with FASD, some of which are included in the above-mentioned diagnostic criteria, such as deficient brain development, congenital malformations, and neurobehavioral impairment. Some of the typical comorbidities reported can be directly linked to teratogenic effects of alcohol such as developmental disorders and sensory system problems (hearing impairment and ophthalmological diagnoses). Other comorbidities can be considered secondary and possibly preventable problems, for instance, behavioral problems such as conduct disorder and substance use disorders [8].

Adolescents and adults with FASD have a high risk for disrupted education and being institutionalized either in prison or facilities for persons with psychiatric and substance use disorders, but an early diagnosis of FASD and a stable living environment may have the potential to reduce the risk for these adverse outcomes [9,10]. It has been shown that FASD is associated with significant economic costs in the USA and Canada [11]. In Sweden, where the social and health care system is similar to the Finnish system, the estimated mean annual cost is EUR 76,000 per child (0–17 years) with FAS [12].

### 1.5. Motivation for the Present Study

There is currently a lack of knowledge and understanding regarding the emergence and evolution of problems associated with FASD during an individual’s development and what the needed interventions are, their cost, and when they should be introduced.

Because there is a severe problem of underdiagnosis, there is a need for knowledge of the typical age-specific patterns of problems associated with FASD to be able to better identify persons with possible FASD for evaluation and planning of interventions. Because early intervention and prevention of secondary problems is typically more cost-efficient than trying to mitigate already emerged and chronic problems, we also need to anticipate the trajectories of typical problems. To reduce the societal costs associated with FASD, we also need to know the cost structure.

In Finland, there is an extensive and reliable unified electronic patient register within each hospital district. In addition, the Patient Data Repository (Kanta) is a national information system service for archiving electronic patient data produced in the healthcare service. The Client Data Act obliges all public and private healthcare providers to join the Kanta. Furthermore, all medication prescriptions are saved in the Prescription Centre, which is a centralized database for prescription data as a part of the Kanta. The data stored in the Kanta archive are accessible by healthcare professionals and can be used for gathering a patient’s health history during the clinical evaluation. This enables performing detailed studies based on electronic patient registers.

In Finland, FASD is typically diagnosed in pediatric neurology outpatient clinics in hospitals and sometimes in pediatric or adolescent psychiatric outpatient clinics or inpatient wards in hospitals. In both pediatric neurology and psychiatric clinics, patient entries are typically very detailed and include a wide range of information concerning patients’ health and family history, developmental history, results of all assessments and evaluations by a multidisciplinary team, as well as reports from therapies, day care, and schools. They also include recommendations for rehabilitation, disability allowance, and special school arrangements. Patient register data are therefore not limited to health information but allow for more complete analysis of problems, interventions, and their costs.

### 1.6. The Aims and Type of the Study

In this detailed retrospective descriptive electronic patient register-based chart review study of all patients diagnosed with FASD in one Finnish central hospital, we wanted to discover the following:

1. What were the most common diagnoses and problems in everyday life for those diagnosed with FASD within four age groups: infancy, preschool age, primary school age, and adolescence?
2. What were the typical interventions in different age groups?
3. What were the estimated additional costs associated with problems and interventions in different age groups?

## 2. Data

### 2.1. Study Population Definition

In Päijät-Häme Central Hospital, which is a secondary-level hospital in southern Finland with a catchment population area of 220,000, we included all persons who had been patients (either as inpatient in any ward or as outpatient of any hospital clinic) in the Päijät-Häme Central Hospital between 2010 and 2022 and had one of the following ICD-10 diagnoses:

- P04.3 (Fetus and newborn affected by maternal use of alcohol);
- Q86.0 (Fetal alcohol syndrome or Partial fetal alcohol syndrome);
- F83 (Mixed specific developmental disorders) or F81.3 (Mixed disorder of scholastic skills) or F90 (ADHD) and had FASD or ARND listed as a written sub-diagnosis, or maternal alcohol use during pregnancy was listed as the etiology for developmental disorder.

Available information was collected from the patient register between 1 October 2021 and 30 September 2022. A total of 55 persons were included. All those with the initial diagnosis of P04.3 had later been diagnosed as having some type of FASD.

There was not enough reliable information about the quantities or timing of alcohol exposure to include it in analysis. In eight patients, alcohol exposure was reported to be accidental due to late recognition of pregnancy, and in them the reported exposure varied from heavy exposure in early pregnancy to repetitive moderate exposure during the whole pregnancy because the pregnancy was not recognized before labor began. In others, the diagnosis of maternal alcohol use disorder was considered as the reason for alcohol exposure. In some patients, the prenatal alcohol exposure had been recognized and addressed already during prenatal care, but the information concerning timing of recognition, interventions provided during pregnancy and their effectiveness were not available. In others, the information about alcohol use during pregnancy or maternal alcohol use disorder was postnatally retrieved from the mother, close relative or social worker. We did not have access to maternal health records.

In Finland, out-of-home placement is considered as the last resort when all other support interventions for the family have failed, or when there is a severe acute crisis in the family and milder interventions are not sufficient. Still, the majority of the patients diagnosed with FASD were in foster care or had been adopted by the time of the first evaluation in the hospital. Maternal alcohol use disorder is typically at least one of the reasons why the child is taken into custody by child protection services and placed out of home.

### 2.2. Data Coverage

**Figure 1:**
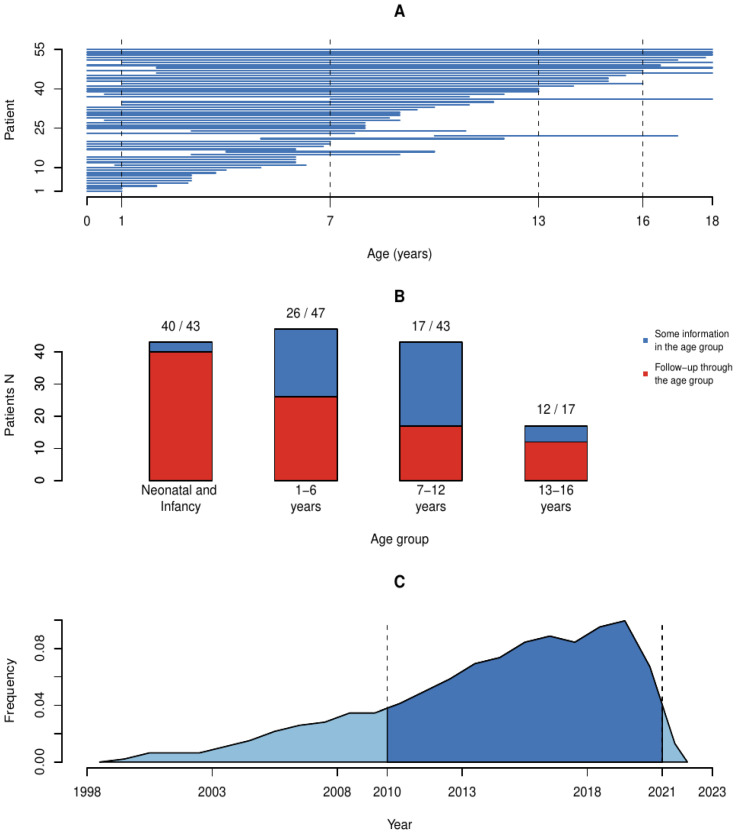
The patient follow-up times and number of persons with information available in each age group. The follow-up times varied. (**A**) Lines show the known period each patient was living in the hospital district. (**B**) Number of patients in each age group, red color indicating those with complete follow-up through the age period and blue those with partial follow-up. (**C**) Follow-up-time density by year shows that 79.7% of follow-up times occurred between 2010 and 2021.

We determined that the data collected from hospital records were limited because full hospital records were available only for the period when the patients were living at the Päijät-Häme hospital district, leading to varying lengths of follow-up times (Figure 1). Data collected from pediatric neurology unit records were comprehensive. On the contrary, information concerning the time before first referral to the pediatric neurology unit depended on the level of detail of information in the referral. Subsequent post-follow-up data after the last visit in the pediatric neurology unit were often scarce.

The electronic patient register was introduced in 2010, which means that full information before 2010 was available only in paper records. The most important cause of missing information was out-of-home placements and the subsequent moving of patients from one hospital district to another. Even with the national Kanta records, some patients were unavailable for follow-up because they moved out of the hospital district and the research permit did not allow searching the Kanta for information of patients who were no longer living in the hospital district. The Kanta was introduced between 2010 and 2014 and the use of electronic prescriptions was not mandatory before 2017. This means that even for patients living in the hospital district, older information from other hospital districts was not available. For those who moved from other districts or countries to the research district, referral information was often incomplete. Further, foster parents could not help with missing history, because medical information is typically not disclosed to the foster parents by the authorities.

There was very limited information available during adolescence, especially between ages 16 and 18, because pediatric neurology clinics and pediatric clinics treat and follow patients only up to 16 years of age. To have more complete information, we decided to limit analysis to 0–16 years of age.

## 3. Methods

### 3.1. Data Collection

From the unified electronic health record system used in the Päijät-Häme Central Hospital, of those 55 persons diagnosed with FASD, we collected information from birth and all hospital visits either as inpatient or outpatient, developmental assessments, rehabilitation interventions, long-term medications for chronic conditions, referrals from basic health care, support arrangements and their levels at kindergarten and school. Each type of information was analyzed descriptively divided for age groups to determine typical problems and interventions at each age. The data were collected mainly by one person (LMJ); some information was collected by HS.

#### 3.1.1. Birth Information

The collected birth information included gestational age, birth measurements of length, weight, head circumference and their standard deviation Z-scores, Apgar scores, pH of umbilical artery, treatment in neonatal intensive care and surveillance unit (NICU), and neonatal diagnoses. Available information was collected concerning maternal use of tobacco, drugs—both legal and illegal—and alcohol during pregnancy.

#### 3.1.2. Diagnosis-Related Information and Medications

We collected all hospital outpatient and inpatient examination and treatment data including main diagnoses, expensive examinations such as brain MRI, sleep–wake EEG, molecular karyotype/chromosomal microarray and fragile-X-examination, patient age at the visits and prescribed long-term medications. We collected the type of FASD (FAS, PFAS, or ARND) and age at the diagnosis. Main diagnoses in each age group were analyzed descriptively to determine most common diagnoses indicating typical age-specific problems in the study population.

#### 3.1.3. Developmental and Rehabilitation Information

Developmental information that was collected included the test results of the assessments by occupational therapists, speech therapists and neuropsychologists, as well as rehabilitation recommendations at each age. Developmental and rehabilitation history before first assessment in the pediatric neurology clinic was collected from parents and referrals from primary health care services.

#### 3.1.4. Educational Information

We collected educational special needs support arrangements and the level of support recommended and received separate information for kindergarten, primary school grades (7–12 years) and middle school grades (13–16 years). The frequencies of each support level in each age group were analyzed. The learning objective level according to the type of syllabus used (regular or individualized) was collected separately as an indicator for severity of learning difficulties.

#### 3.1.5. Family Information

We also collected the family situation history according to information collected in patient record texts, often by a hospital clinic’s social worker. In case of out-of-home placement, we extracted the age at first known out-of-home placement and, when relevant, the age when the child was returned to home or adopted, as well as the number and types of placements (foster family or some type of institutional placement), when available.

#### 3.1.6. Forming of Age Groups

We divided the collected information about problems and interventions into four different age categories: infancy (0–11.99 months), preschool age (1.0–6.99 years), primary school age (7.0–12.99 years) and adolescence (13.0–15.99 years).

### 3.2. Cost Estimation Methods

#### 3.2.1. Selection Process of Cost Estimation Factors

For cost estimations, we included only additional costs that are not part of a regular childhood. That is, we considered that health care, education, and social assistance costs estimated for healthy children with normal development living at home with birth parents represent the baseline costs for comparison to children with FASD, thus excluding those costs in cost estimation. We used regular health economic macro-costing methods with available average prices for each cost type. These included Diagnosis-Related Group (DRG) prices and statistical average costs and prices in the hospital catchment area for out-of-home placements and rehabilitations and for each type of special needs education costs. We estimated costs for medication and disability allowance using available national prices, because these are not area-dependent. In Finland, we have publicly available detailed statistics of cost and prices in all areas of public expenditure.

Because DRG prices lack accuracy in individual cases, it has been recommended that additional cost analysis should be performed for specific therapeutic areas with high costs [13]. That is what we aspired to do by examining individual patient records and adjusting costs that deviated significantly from the diagnosis-based DRG price by using DRG ward-day pricing multiplied by the stay length instead of diagnosis-based DRG price in the case of exceptionally long hospital stays. Specific explanation is given in Section 3.3.1 Hospital costs. The same type of correction was performed for those patients with known lengths of exceptionally expensive institutional care.

#### 3.2.2. Index Correction and Discounting

Because we did not have access to actual unit costs (original nominal past pricelists) except for out-of-home placements, we used the available pricelists from 2021. First, to obtain an estimated nominal cost of each service for each year, we corrected the unit cost based on the price index of public expenditure for social services and healthcare of local government finances by function area [14]. The index includes technological and medical advances and policy changes, and the changes they have caused in the prices of public health care and social services during the follow-up years. Having thus estimated all nominal costs, we corrected them with the general price index of Finland [15] to express them in comparable real values using 2022 euro currency as constant. For the out-of-home placements, only the general price index correction was used when nominal past prices were already available for years 2006–2020. For years before 2006 and years 2021 and 2022, the price index of public expenditure for social services and healthcare was applied first to obtain nominal prices before general price index correction, as with other prices.

#### 3.2.3. Estimated Cost Types

Estimated costs included:

(1) Hospital costs, including both outpatient clinic visits and inpatient ward stays;
(2) Out-of-home placement costs, both in foster families and in institutional placements (not including hospital stays, which are under hospital costs);
(3) Disability allowance costs, which is money paid by the Social Insurance Institution when a child has a chronic illness or a disability and has greater than normal need for care and attention;
(4) Rehabilitation costs including physiotherapy, speech therapy, occupational therapy, neuropsychological rehabilitation and psychiatric therapies;
(5) Special needs education costs (average schooling costs were subtracted);
(6) Long-term medication costs for regularly used prescription medication (not including asthma and allergy medications, which are common also in general population).

### 3.3. Cost Estimations

See Appendix A and Table A1 for the list of prices before index correction. The results were analyzed using custom scripts in R 4.2.1 [16].

#### 3.3.1. Hospitalizations

Hospitalization and hospital visit costs were estimated using available DRG prices for each main diagnosis listed in hospital clinic outpatient visit or hospital ward inpatient stay during each age group. We used the 2021 price list for services in the Päijät-Häme area [17]. All adolescents diagnosed with F94.1 (Reactive attachment disorder), F32.9 (Depressive episode) and F50.0 (Anorexia nervosa) needed long inpatient treatment in the adolescent psychiatric unit, which is significantly more expensive than the DRG price based on adult patients. For a more accurate estimation of those diagnoses, instead of using diagnosis-based DRG price, we used the DRG price for adolescent psychiatric ward multiplied by the length of stay. In addition to diagnosis-based DRG prices, we added costs from sleep–wake EEG, brain MRI and molecular karyotyping in hospital costs depending on the age at which they were performed.

#### 3.3.2. Out-of-Home Placements

Out-of-home placement costs were estimated by extracting the years a child was placed out of home and considering the sum of their nominal out-of-home placement costs in the hospital district during 2006–2020 [18,19]. For the out-of-home placements before year 2006, we used the price for 2006 and applied the price index of public expenditure for social services and healthcare of local government finances by function area to obtain nominal prices. The same was performed for years 2021 and 2022 using the price of 2020. For the patients with known length of specialized and thus expensive institutional placement, the annual cost difference between institutional placement and average cost was estimated using the more specific pricing data available for the six largest cities in Finland [20]. The resulting institutional cost multiplier was 1.5 times the nominal cost. The out-of-home placement was considered to have ended when a child was returned to their birth family or adopted. Index correction using consumer price index was then applied for the costs according to their years, as in other types of costs.

#### 3.3.3. Disability Allowance

Disability allowance costs were estimated by multiplying the presence and level of disability allowance and time in years in each age period. Because foster parents do not typically know the disability allowance level their fostered child is granted, we estimated the disability allowance levels according to diagnoses, rehabilitations, and clinical experience from other patients with similar problems, using the Social Insurance Institution of Finland’s (SII; “Kela” in Finnish) disability allowance criteria and amounts for 2021 [21].

#### 3.3.4. Rehabilitation

Rehabilitation costs were estimated using the typical rehabilitation schemes in our hospital district, because the exact number of realized rehabilitation sessions was not easily available. In Finland, rehabilitation for severe developmental disorders (intensive medical rehabilitation) is financed via SII and requires a diagnosis by hospital clinic indicating a specific developmental disorder. Rehabilitation for mild or intermediate difficulties and early interventions before having specific developmental disorder diagnosis are arranged via either hospital or basic health care and financed by the hospital district. If a patient underwent a rehabilitation listed in patient records without a diagnosis of specific developmental disorder, it meant that the rehabilitation was financed by hospital district, and cost was estimated using the pricelist for services in Päijät-Häme area 2021 [10] and average number of rehabilitation sessions of each therapy type when arranged by hospital district. For those with specific developmental disorder diagnoses, therapies were financed via SII. Their rehabilitation costs were estimated as averages for the type of rehabilitation in the hospital district area from SII Procurement decisions of the Southern Insurance District 2018 [22] and SII Rehabilitation services arranged by Kela statistics [23] and the typical recommended number of therapy sessions and duration of therapy in years for each type of developmental disorder in our pediatric neurology clinic.

#### 3.3.5. Special Needs Educational Support

Special needs educational support costs were estimated according to the level of special support listed in kindergarten (5–6 years), primary school (7–12 years) and middle school (13–16 years). Kindergarten-age children had included costs only in case of compulsory extended education, which means two years of kindergarten with special education, and only the costs of additional kindergarten year were included. The mean costs for a typical student without extra support was subtracted from all special needs education costs. Costs were retrieved from the report on financing of educational and cultural provision 2020 from the Finnish National Agency for Education [24] and cost statistics from the Finnish National Agency for Education [25]. Listed and possible support included, from most to least expensive support level:

1. Entry in specialty school for disabled with extended compulsory education and individualized syllabus; very high adult/student ratio; at this support level, taxi transfers and morning and afternoon care are included in total cost;
2. Extended compulsory education in a small special needs class and with individualized syllabus, but situated in regular school; high adult/student ratio;
3. Special needs education and special support in small class without extended compulsory education; general or modified syllabus; higher than average adult/student ratio;
4. Special support with inclusion and integration to mainstream classroom;
5. Intensified support in mainstream classroom.

Additionally, we extracted the information and estimated costs for possible morning and afternoon care for intellectually disabled students after first grade for those attending regular schools. Further, costs for taxi transfer were estimated according to the same guidelines. We considered special needs educational cost information missing if the patient was not old enough to have attended one full year of the educational stage in the age group.

#### 3.3.6. Medication

Long-term medication costs were estimated using the prices for a typical dosage and medication for each medication type according to patient’s age and typical medication follow-up costs including laboratory tests and medication control health checks for each type of medication. Diagnosing costs were not included because they were included in the hospital visit DRG price. In Finland, prescription medications have the same prices in all pharmacies. Only medications that were prescribed in hospital clinics and used for at least a year were included. Because medications for asthma are also very common in the general population and prescribed in primary health care for older children, they were not included.

#### 3.3.7. Calculation Methods and Assumptions Used in Estimations

For each cost category, we calculated the sum of the known estimated costs for each patient within the age groups. If the patient was living in the hospital district during certain age group, but did not have hospital visits, known rehabilitation or known long-term medication, known special needs education services or out-of-home placement mentioned in patient register data during that age period, we assumed that they did not have those services during that period, thus resulting in zero costs for the patient within the age group. If the patient was not living in the hospital district during a certain age and the entry was missing or stated not available, we set the costs as not available.

Because the out-of-home placement data and special education data were not always synchronous with hospitalization data, we recorded the corresponding follow-up time for them separately for each patient according to the entries in the hospital records. To accommodate the left and right censoring, we also calculated the follow-up time at each age group separately. If a patient did not have a follow-up at a certain age, the follow-up time at the respective age was 0. Thus, even when the age group size varied at different calculations, the annual costs were consistent.

For the mean annual costs, we first aggregated the age-group-specific costs by the cost category. Next, the sum of each cost category was divided by the sum of the follow-up time for the respective age group and the cost category. Similarly, we calculated the total mean annual cost by summing all costs within the age group and dividing the sum by the follow-up time. We also separately calculated mean annual costs for those with complete follow-up through each age group.

To determine the cumulative costs, we calculated the sum by two methods: First, we selected the patients with at least 15 years of continuous follow-up time and complete records at each cost category. In this group, we calculated the cumulative sum directly for each person through the age groups. The other method consisted of selecting at each age group the patients with complete follow-up time through the respective age. We calculated the cumulative sum by adding the mean costs of each age group to the mean cost of the previous age group.

## 4. Results

Study population descriptive statistics can be found in Table 1.

### 4.1. Infancy, Age under 1 Year (Diagnoses and Interventions n = 43, Complete Follow-Up Time n = 38)

#### 4.1.1. Diagnoses

Table 2 shows the five most common diagnoses. Full birth and neonatal care information was available for 38 persons who were born in our hospital. The majority (31/38, 81.6%) had at least one neonatal diagnosis. During the first year, 14/43 children (32.6%) were diagnosed with at least one congenital malformation other than FAS (Q86.0). Congenital heart defect (CHD) was diagnosed in 8/43 (18.6%): three atrial septal defects, three ventricular septal defects, one atrioventricular septal defect and one child was operated on for patent ductus arteriosus. The second most common congenital malformation was microcephaly diagnosed in 6/43 (13.9%), though 19/44 (43.2%) had a birth head circumference at −2 SD or smaller, indicating microcephaly. Another eight congenital malformations were unique. Four persons had two separate malformations and five persons had three separate malformations.

#### 4.1.2. Interventions

The main interventions (Table 2) during infancy were treatment in the neonatal intensive care and surveillance unit (NICU) (29/38, 76.3%), physiotherapy for motor development and muscular tone problems in 29/43 (72.5%) and out-of-home placement in 23/43 (57.5%). Four of those placed out of home were adopted before their first birthdays. Hospitalizations for infections were common (14/43, 32.6%), and five (11.6%) had three or more hospitalizations for infections during infancy. The most common hospitalization main diagnosis was otitis media (10/43, 23.2%).

#### 4.1.3. Costs

**Figure 2:**
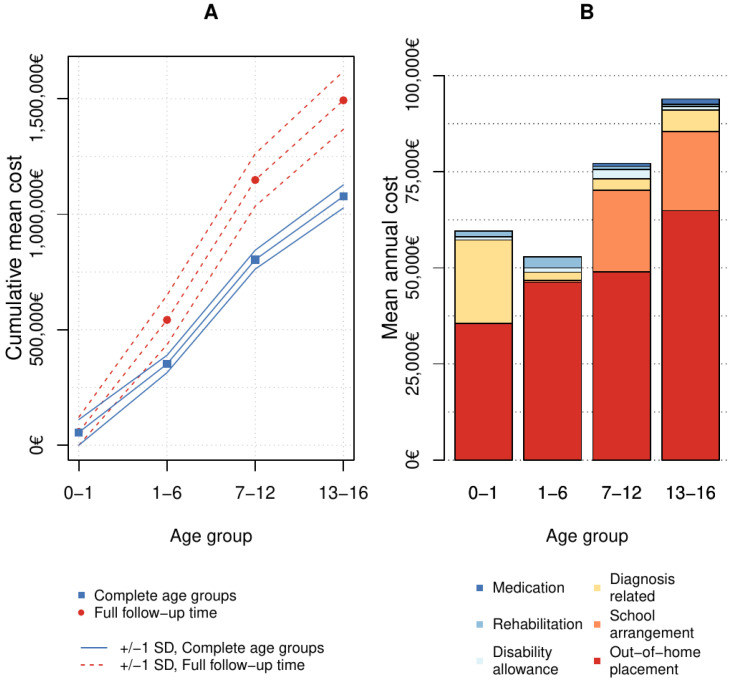
Cost accumulation and distribution per age group. The mean additional costs for 0–16 years that were not part of regular childhood costs that reached EUR 1,077,000, using regular childhood costs as the baseline and using year 2022 euro currency. (**A**) Using all patients with complete follow-up within each age group (<1 years n = 38, 1–6 years n = 26, 7–12 years n = 17 and 13–16 years n = 12), the mean cumulative costs start to increase more steeply during school years (solid blue line). However, using the 10-patient subset with complete information for at least 15 full consecutive years, the cumulative costs increase more linearly at each age group (red dashed line), reaching 1.5 million euros per person. (**B**) When calculating the costs per person years, out-of-home placement formed the largest proportion of costs in all age groups.

Estimated mean annual costs, their medians and interquartile ranges are presented in Table 3. The age-specific cost distribution is shown in Figure 2. Most diagnosis-related costs during infancy were caused by treatments in the NICU. Prematurity, neonatal abstinence syndrome and congenital malformations caused long NICU treatments. Out-of-home placements caused significant costs already during infancy. Other types of costs were low. The first-year mean additional costs compared to a healthy population for those with complete follow-up were EUR 55,500 rounded to the nearest full hundred.

### 4.2. Preschool Age (1–6 Years, Diagnoses and Interventions n = 45, Complete Follow-Up Time n = 26)

#### 4.2.1. Diagnoses

In this age group, developmental delays were typical reasons for hospital outpatient clinic visits. (Table 2). In addition to isolated impairments of motor and speech development, 17/45 (37.8%) had a diagnosis indicating impaired global development, either F83 Mixed developmental disorders or F70 Mild intellectual disability. Nearly a third (14/45 31.1%) had at least one ophthalmological diagnosis, of whom 13/14 (92.9%) had H50 Strabismus and/or H53.0 Amblyopia ex anopsia (“lazy eye”), two had a congenital eye or eyelid malformation. Otitis media requiring otorhinolaryngologists evaluation continued to be common also in this group, as in infancy. Psychiatric diagnoses started to increase towards kindergarten age. The most common psychiatric diagnoses were: F90 ADHD in six (13.3%), F94.8 Other childhood disorders of social functioning in four, and F94.1 Reactive attachment disorder in three.

#### 4.2.2. Interventions

Nearly all (41/45, 91.1%) had received at least one form of therapy, and occupational therapy was the most common (39/45, 86.7%). For those 40 assessed by an occupational therapist, fine motor coordination was documented as below normal (<−2 SD) in 36/40 (90%), sensory processing was below normal in 34 (85%), and static balance was below normal in 33 (82.5%). Gross motor abilities were below normal in 19 (47.5%).

**Figure 3:**
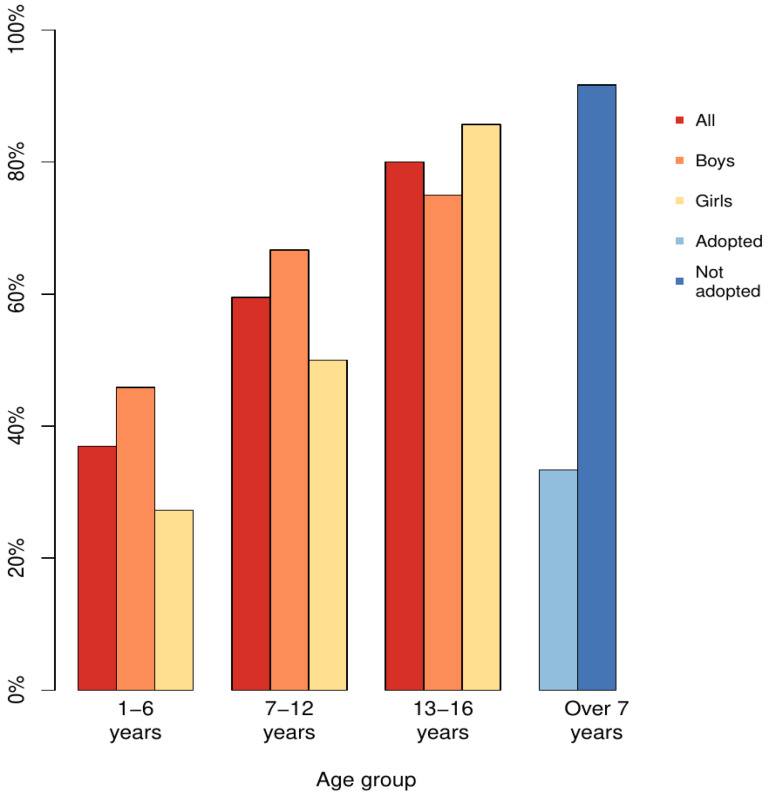
Proportion of persons who have been in psychiatric unit care increased at each age group. For comparison between adopted and non-adopted persons, to obtain comparable groups, data for everyone over 7 years old at the time of data collection were combined.

At this age, 17/45 (37.8%) were placed out of home in addition to the 23 placed already before. Three children returned to their birth families and two were adopted during this age. At the age of 6 years, 35/45 (77.8%) were in out-of-home placements. All those who were returned home continued to be surveilled by child protection services. As many as 17 (37.8%) needed child psychiatric care (Figure 3)

#### 4.2.3. Costs

Although rehabilitation costs were at their highest level before school age, total mean annual costs were at their lowest during preschool age. For those with complete follow-up time, 1–6 years mean annual costs were approximately EUR 49,300 (Table 3), (Figure 2).

### 4.3. Primary School Years (7–12 Years, Diagnoses n = 37, Complete Follow-Up Time n = 17)

#### 4.3.1. Diagnoses

During primary school, attention problems were described in patient records in 36/37 (97.3%) while 25 (67.6%) were diagnosed with attention-deficit hyperactivity disorder (ADHD). At this age, 25 (67.6%) had a diagnosis indicating impaired learning abilities (F81, F83 or F70)—see Table 2.

Psychiatric diagnoses other than ADHD were diagnosed in eight (21.6%) patients, of which two had reactive attachment disorder (F94.1) and two had disinhibited attachment disorder (F94.2).

#### 4.3.2. Interventions

**Figure 4:**
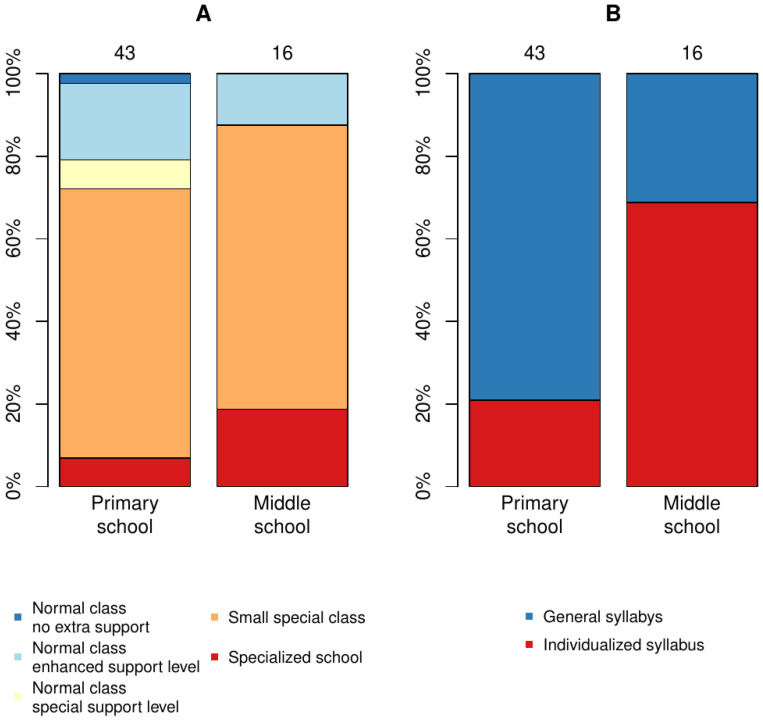
Support level in each school stage. (**A**) The distribution of increasing support levels for school ranging from no extra support in normal class to specialized school for students with significant disabilities. (**B**) Learning objectives. In Finland, the syllabus may be individualized if a student already has a special support level and even with extra support is not able to complete the regular education syllabus.

Psychostimulant medication was prescribed for 24 (64.9%) patients. Regarding the support received or planned for school (Figure 4), only one child did not have any extra support during primary school. Psychiatric care prevalence increased in primary school years (Figure 2). Three patients were placed out of home for the first time during primary school age.

#### 4.3.3. Costs

Costs for special needs education and disability allowance were high at primary school age (Table 3) (Figure 2). Somatic hospital costs were at their lowest at this age, but psychiatric costs were rising. For those with complete follow-up, mean annual costs were approximately EUR 75,300.

### 4.4. Adolescence (13–16 Years, Diagnoses n = 15, Complete Follow-Up Time n = 12)

#### 4.4.1. Diagnoses

Of the middle-school aged adolescents, 12/15 (80%) had a diagnosis indicating impaired learning abilities, either Mild intellectual disability (F70) or Developmental disorders of scholastic skills F81. The majority (9/15, 60%) had dual diagnoses of cognitive impairment and mental health problems, and the other 6 (40%) were diagnosed with either cognitive impairment or mental health problems. The most common main diagnosis in psychiatric unit visits was mild intellectual disability with significant impairment of behavior (F70.1) in five patients. They had symptoms that are typical for conduct disorder, but that was not diagnosed because of intellectual disability. Other non-unique psychiatric diagnoses were depressive episode (F32.9) in three patients, anxiety disorder (F41.9) in two, and reactive attachment disorder (F94.1) in two.

#### 4.4.2. Interventions

The majority (12/15, 80%) were patients in the adolescent psychiatric unit. Those diagnosed with reactive attachment disorder, depressive episode and one with eating disorder (F50) all received long inpatient psychiatric treatments. Most (9/15, 60%) teenagers used at least one regular medication, but only 5 (33.3%) continued psychostimulant medication for ADHD symptoms, although 10 (66.7%) still had ADHD listed as a diagnosis during this age.

Support level in school (Figure 4) was high for middle school; only two persons studied in normal classrooms during middle school, and all received extra support.

#### 4.4.3. Costs

Psychiatric hospitalizations and institutional out-of-home placements increased costs and medication costs were at their highest level during adolescence (Table 3) (Figure 2). Mean annual costs for those with complete follow-up time were approximately EUR 91,200.

### 4.5. Results of the Whole Childhood (0–16 Years)

#### 4.5.1. Etiological Examinations Performed

Brain MRI was performed in 18/55 (32.7%) cases: 12/32 (37.5%) with FAS, 5/17 (29.4%) with PFAS and 1/6 (16.7%) with ARND. Of those who had brain MRIs taken, 10/18 (55.6%) had findings listed in their neuroradiological reports, of which some could be classified as incidental findings or normal variants. There were 4/18 (22.2%) cases of wide/enlarged cisterna magna or prominent retro cerebellar cerebrospinal fluid space, 2 (11.1%) with agenesis or dysgenesis of corpus callosum, 2 (11.1%) had hypoplastic midbrain structures (one of them also had agenesis of corpus callosum), 2 (11.1%) had apparent microcephaly without structural anomalies, and 1 (5.6%) had wide perivascular spaces.

Sleep–wake EEG registration was performed on 15/55 (27.3%): 10/32 (31.3%) with FAS and 5/17 (29.4%) with PFAS. The reasons for EEG were suspected seizures or suspected developmental regression. Of those EEG registrations, 9/15 (60%) were classified as abnormal. Interictal epileptiform discharges were detected in 6 (40%), but only 2 (3.6%) were diagnosed with epilepsy that required medication. Three (20%) had slow background activity.

#### 4.5.2. Non-Age-Specific Somatic Findings

Infections were common even after infancy; 16/55 (29.1%) had at least one hospital visit for recurrent or complicated middle ear infections, 6 (10.9%) had three or more urinary tract infections, 1 had chronically elevated creatinine and cystatin c levels indicating kidney dysfunction but normal kidney ultrasound results, and 5 (9.1%) had tonsillectomy/adenoidectomy performed. Some endocrinological and autoimmune problems were also noticed. Three received growth hormone treatment for short stature and growth hormone deficiency. One had juvenile diabetes mellitus, and two had recurrent hypoglycemic and reactive hyperglycemic episodes without diabetes. One had celiac disease, one had hyper-IgA without apparent cause, and two had significant hyperprolactinemia associated with risperidone medication.

#### 4.5.3. Adopted Compared to Those Living in Birth Families or Foster Care System

Those 8/55 (14.5%) who were adopted more often had a full FAS 6/8 (75%) diagnosis compared with the others, lower average birth weight (2180 g) and smaller latest average head circumference Z-score (−2.6 SD, SD 1.0). All but one of the adopted lived in their adoptive families since infancy; three first as foster children. Mean age at the time of data collection for the adopted was 13.6 years (7–24 years, SD 5.1). Comparing those who were adopted to others who were not adopted but were also at least 7 years old at the data collection (n = 35, age 7–28, mean 13.1 years, SD 4.8), fewer adopted (2/8, 25%) had had psychiatric contact before the age 16 years than non-adopted (32/35, 91.4%) (Figure 2). Those who were adopted did not differ from non-adopted children according to cognitive function or support needed for education nor the use of psychostimulant medication.

#### 4.5.4. Cumulative Additional Costs for the Childhood (0–16 Years)

The majority (78.5%) of the estimated mean cumulative total costs of EUR 1,077,000 for 0 to 16 years using those with complete follow-up within each age group (<1 year n = 38, 1–6 years n = 26, 7–12 years n = 17, and 13–16 years n = 12) occurred from long-term out-of-home placements (Figure 2). For those 10 persons with full continuous consecutive information for at least 15 years available during their childhood, the mean total cost was EUR 1,493,200 per person. Special school arrangements caused most costs for those growing up in birth or adoptive families. Rehabilitation and health care costs were at their highest during early years, except psychiatric costs that increased with age.

## 5. Discussion

This study clarified the phenotype of FASD and its evolving pattern of health care needs and their associated costs during childhood. Typical problems in each age group and high costs among children with FASD were in line with many previous findings. There is a need for a syndromic approach to diagnosis and multidisciplinary follow-up in FASD. When we know to search for typical problems at each age, we can move from treating symptoms towards prevention of secondary problems. Early diagnosis, counseling for parents and caregivers, psychological support and realistic expectations for the children have been shown to improve their long-term prognosis [9,27].

High incidence of neonatal problems, including prematurity and congenital malformations in children who later become diagnosed with FASD, has been described many times before [28]. It underlines that the maternal history of alcohol use is important pediatric information and should be available to midwives, obstetricians, and neonatologists. The 18.6% prevalence of CHD was high, but comparable with some earlier studies on persons with FAS [29] and significantly higher than 0.9% in the whole population [30]. FASD should be recognized as a syndrome associated with high risk for cardiac abnormalities that warrants early cardiac examination.

Learning during early development happens by observing, interacting, and experimenting with the environment. For that, a young child typically uses eyesight, hearing and other sensory input, and their ability to manipulate objects by hand. Importantly, this study showed that eyesight, hearing, sensory processing and fine motor skills are often at least temporarily impaired in small children with FASD. Early recognition, treatment and adequate timely rehabilitation of these problems may improve the developmental trajectory that now often seems poor.

The high prevalence (31.1%) of early onset visual problems, especially strabismus (26.7%) and amblyopia, was lower than in a study on internationally adopted persons with FASD [31], maybe because only those with apparent symptoms had been assessed by an ophthalmologist, but it is significantly higher than 2% prevalence of strabismus in the whole population [32]. A spectrum of ophthalmological problems has been associated with FASD [33] and even diagnostic tools consisting of ophthalmological findings have been developed [34,35]. All children with FASD should have an ophthalmologic assessment because unrecognized visual problems increase risk of poor school performance [36].

Recurrent or complicated middle ear infections that cause intermittent conductive hearing impairment in prenatally alcohol-exposed children has also been described before [37,38]. Otitis media as the main diagnosis for hospital visits typically means a need for tympanostomy, and before that a child’s hearing could have been reduced because of adhesive otitis for several months in the age period of fast language acquisition. Children with FASD should have a hearing examination and be evaluated for glue ear [39].

Almost universal sensorimotor problems before school age—sensory processing was below normal in 85% and fine motor skills in 90%—support the finding that prenatal alcohol exposure alters sensorimotor network in the brain [40]. In addition to poor executive functioning, sensorimotor problems have been proposed to be one explanation for poor adaptive skills and school performance [41].

Although the majority of the study population required physiotherapy during infancy and speech therapy during preschool age, significant catch-up in both gross motor skills and speech was common with rehabilitation. Inverse trajectory was seen in learning abilities. Although 79.1% started school according to the regular syllabus, increasing support was gradually needed, and in middle school, only 31.2% was able to follow the regular syllabus and all received extra support. Learning problems became more significant when demands for executive skills and abstract thinking increased after the first primary school years. Learning support should be provided from early on, instead of waiting for the child to fail before providing the necessary support.

In addition to cognitive difficulties in FASD, adaptive problems that impair everyday functioning tend to become more apparent when socioenvironmental requirements increase with age [10]. The prevalence of intellectual disability among teenagers in our study population (53.8%) was higher than 17% in a US study [9] but comparable with other studies on persons with FAS [42,43]. This may reflect the reality that mainly those with severe problems have been diagnosed with FASD in Finland. High percentage of ADHD and learning disabilities in FASD [8] was confirmed in this study. Psychostimulant medication for ADHD was started before middle school in 64.9%, but half discontinued using it, indicating that psychostimulant medication may not be as beneficial in persons with FASD as it is with ADHD caused by other factors, or adverse effects may be more significant in persons with FASD.

Secondary psychiatric problems increased with age, especially during puberty, and 80% of adolescents received psychiatric treatment indicating deficient early support and possibly a poor postnatal living environment, although 41.8% had been placed out of home already before the age of one year. Being adopted appeared to be associated with lower secondary psychiatric problems, supporting earlier findings that a stable family environment from early age is beneficial for mental wellbeing [9], because 7/8 of the adopted in this study had been living in the same family since infancy. However, in this study, even early adoption did not diminish the ADHD-like symptoms and the need for psychostimulant medication, nor did it reduce support needed in school. It has been shown earlier that a stable postnatal environment does not mitigate the neurobiological disorder caused by prenatal alcohol exposure nor are typical symptoms caused solely by an adverse postnatal environment [44].

MRI findings among those who had brain MRI scans may overestimate the prevalence of abnormal MRI results, because the majority had not been scanned. However, the findings indicated that 22.2% had prominent retrocerebellar CSF space indicating cerebellar and midbrain hypoplasia and 11.1% had dysgenesis of corpus callosum, which is in line with previous research [45,46] showing that cerebellar and midline development are particularly sensitive to ethanol toxicity. A brain MRI may support a suspected FASD diagnosis, but it requires anesthesia unless performed during the newborn period or is delayed until school age.

This study confirmed the high societal cost of FASD during childhood, although only part of all societal costs, was possible to collect or estimate from hospital records. The aim was to include only those additional costs that are not present during a typical childhood. We included and estimated costs for special health care, meaning hospital inpatient stays and outpatient clinic visits, rehabilitation, out-of-home placements, regular long-term medications, disability allowance and extra support needed for school. Naturally, all children cause significant societal costs, such as primary health care, day-care, short-term medications for common ailments, and schooling costs, and these also may differ in children with FASD compared to the whole population, but those costs were not part of this study and considered as baseline.

Mean annual costs were estimated using the year 2022 prices. They should be interpreted as the costs that significant health and social care needs and interventions for a child with FASD would currently cause for the society in one year in Finland. Mean annual additional costs were age-dependent and varied from EUR 49,000 in the preschool age to EUR 91,000 during adolescence. The mean annual calculated costs for those with full consecutive information available were EUR 93,300 between ages 0 and 16 and significantly higher than for corresponding age group averages. Our totals were close to the EUR 76,000/year estimated in a Swedish study [12]. The small number of those with complete follow-up during school age in the study population may have affected the estimates.

An important finding was that some problems remain undiagnosed even when acknowledged in rehabilitation or patient register texts, which makes register-based or only diagnosis-based research prone to show less issues than there are in reality. For example, only 31.6% had the diagnosis of fetus and newborn affected by maternal use of alcohol (P04.3), although all had been exposed to alcohol. During infancy, only 13.9% had a diagnosis of microcephaly (Q02), although 43.2% were microcephalic at birth. Attention problems were described in all but one during school age, but just 67.6% had a diagnosis of ADHD. In addition, the number of specific psychiatric diagnoses was lower than the number of children who had been treated in pediatric or adolescent psychiatric units. There are probably different reasons for not entering a formal diagnosis in the patient register even when the problem is acknowledged in text. One reason could be not wanting to stigmatize the child or unsettle parents; another reason could be a habit of just entering one diagnosis, even when there are many recognized problems.

### 5.1. Strengths of the Study

Patients were examined in the same unit and the hospital’s full comprehensive electronic patient record information was available. We were able to obtain detailed individual age-specific information of the sequence of problems and interventions including not only diagnoses and medications but also rehabilitations, out-of-home placements, social benefits and special schooling arrangements.

The study population was ethnically homogenous, all but two were of Finnish or other northeastern European origin.

It was possible to estimate typical interventions and their associated costs fairly accurately because the main author (LMJ) of this article conducts clinical work with these and similar patients and is familiar with rehabilitation and the school system in the hospital district.

The majority of those patients in this study who were diagnosed in our hospital during the last five years had been diagnosed with FASD by two of the authors (HS and LMJ), ensuring that the diagnostic protocol was uniform, and patients met all the IOM’s diagnostic criteria for FASD.

All the estimated costs are additional costs that occur in addition to regular childhood costs. This means that all costs estimated in this study are related to significant problems that are not a part of a regular childhood and thus can be interpreted as costs that are sequelae of FASD. For typically developing children living with their birth families without significant chronic medications or health problems that require hospital visits, there would not be any of these estimated costs. No basic health care costs nor basic schooling costs nor easily accessible community support services were included, because they were considered to be part of the baseline.

Combining in a single study clinical data concerning problems and interventions with their estimated costs (including areas of health care, education and social work) enables us to see a more comprehensive picture of the multifaceted effects associated with FASD in different periods of childhood.

### 5.2. Limitations of the Study

#### 5.2.1. Limitations Related to Data and Study Population

The retrospective nature of the study precludes assessments of record accuracy and completeness. Furthermore, the data were originally recorded for treatment and/or administrative purposes, without considering secondary use, such as cost estimation, and therefore was by nature incomplete for our study purposes. This is especially pertinent to estimates derived from coding text, which can therefore be assumed to be conservative.

The study population was small and derived from a single hospital in Finland, and follow-up time and age of patients at the time of neurological assessments and data collection time varied. Because out-of-home placements and adoptions were common, parental and prenatal information were incomplete. We did not have access to maternal health records and there was not enough reliable information about the quantities or timing of alcohol exposure to include in the analysis. Patients often moved in and out of the hospital district and there were changes in living arrangements, which caused a paucity in records. There was very limited information about teenagers. Incomplete follow-up times even within an age group are likely to underestimate the real prevalence of problems and interventions.

The high percentage of children in out-of-home placements suggests that it has probably been easier to diagnose FASD in children who are not living and accompanied by their birth mothers. Most children with undiagnosed FASD are probably living with their birth families and have been diagnosed with a variety of symptoms and comorbidities. The problems observed in early childhood can be linked directly to prenatal alcohol exposure, but with increasing age, the environment may have a more important role. Children who have been placed out of home often experienced inadequate parenting and maltreatment and some of their problems might be secondary to a traumatic postnatal life.

In a clinical data set without a control group, there is always a possibility of bias towards excessive problems, because only those with significant problems are referred for a hospital clinic assessment and are tracked for longer periods of time.

There is a need for repeated studies with larger sample sizes from multiple hospital areas and, if possible, additional data sources, such as national health and social care registers.

#### 5.2.2. Limitation Related to Cost Estimations

As all cost and comorbidity prevalence estimates are based on small numbers, they are subject to substantial random sampling bias and must be treated as approximates. Cost calculations are also conservative estimates because of the retrospective study design; in addition there are types of additional costs that cannot be estimated using patient register data. There is always a significant amount of uncertainty and potential inaccuracy when using macro-costing methods, which tend to underestimate real costs.

We did not have access to actual nominal prices of the past except for out-of-home placements, and using current price lists to estimate past nominal prices may have caused inaccuracy and bias, although the used services were similar during the years.

We had no access to registers of social services. There are some additional societal costs associated with FASD that could not be included, although they also are rare in the regular non-FASD population. There are typically other extensive services provided for a family before out-of-home placement is considered and also when a child has been returned home after an out-of-home placement. Costs occurring because days off work or reduced working hours of parents or caregivers could not be included. Further, neither costs caused by damaging objects nor criminal activities could be included, because of the lack of specific information in patient records, although there were some mentions of criminal or antisocial behavior during adolescence. Juvenile delinquency has been shown to cause high costs in the FASD population in Canada, for example [47].

#### 5.2.3. Limitations Related to Transferability to Other Countries

Services and their related costs differ between countries and even areas. This study represents Finnish health care, social services, and public education services. It is fairly similar to other Nordic countries and comparable to other countries with public health care and social welfare systems. It differs significantly from countries with mainly insurance-based and private services. However, all the problems and needs revealed in this study would cause significant costs in any country. Finland has high efficiency in health care production and a sustainable healthcare system in comparison with other OECD countries [48,49]. In other OECD countries, costs for similar problems may thus be even higher.

## 6. Conclusions

Children with FASD have significant health, psychiatric, cognitive, adaptive and social burdens that are associated with high costs for society. The revealed age-specific pattern of problems in this study could be used as an aid for recognizing persons with undiagnosed FASD and constituting a follow-up protocol. Early diagnosis is important to make sure that they have the necessary assessments and support in all phases of childhood to minimize secondary problems and high long-term costs. Young children with suspected FASD need to be examined by a pediatric neurologist, cardiologist, ophthalmologist and otorhinolaryngologist, and they also need a developmental assessment by an occupational and speech therapist and psychologist. Both cognitive and adaptive functioning should be evaluated in person with FASD. Because many problems become apparent only with age, and because the pattern of difficulties evolves, it is important to keep following those with known or suspected prenatal alcohol exposure, even when initially they might seem to do well or catch up with normal development. Support for a stable living environment is important for as smooth a development as possible.

## Figures and Tables

**Table 1:** Study population description.

	FAS, n = 32	PFAS, n = 17	ARND, n = 6	All, n = 55
Sex (male), n (%)	20 (62.5%)	8 (47.1%)	3 (50%)	31 (56.4%)
Tobacco exposure, n (%)	20 (62.5%)	10 (58.8%)	4 (66.7%)	34 (61.8%)
Known drug * exposure, n (%)	7 (21.9%)	7 (41.2%)	1 (16.7%)	15 (27.3%)
Gestation at birth mean (SD)	38.0 weeks (3.1)	39.5 weeks (1.8)	37.5 weeks (4.9)	38.4 weeks (3.1)
Birthweight mean (SD)	2459 g (677)	3138 g (376)	3041 g (1023)	2735 g (720)
Birth length mean (SD)	45.0 cm (3.5)	48.3 cm (1.4)	47.3 cm (5.4)	46.3 cm (3.6)
Birth head circumference mean (SD)	32.0 cm (2.0)	34.5 cm (1.5)	32.0 cm (3.2)	32.8 cm (2.3)
Born abroad, ** n (%)	5 (15.6%)	3 (17.6%)	0 (%)	8 (14.5%)
Adopted, n (%)	6 (18.8%)	2 (11.8%)	0 (%)	8 (14.5%)
Placed out of home, n (%)	27 (84.4%)	12 (70.6%)	5 (83.3%)	44 (80%)
Age at first out-of-home placement mean (SD)	1.5 years (2.9)	2.3 years (3.9)	2.1 years (2.3)	1.8 years (3.1)
Multiple (>3) placements or institutional placement, n (%)	7 (21.9%)	6 (35.3%)	1 (16.7%)	14 (25.5%)
Age at FASD diagnosis mean (SD)	5.1 years (5.2)	6.1 years (3.8)	6.8 years (4.4)	5.5 years (4.7)
Age at data collection mean (SD)	11.6 years (5.8) 2–24 years	11.1 years (6.4) 3–28 years	9.0 years (3.9) 3.7–15 years	11.2 years (5.8) 2–28 years
Age < 6 years, n (%)	5 (15.6%)	3 (17.6%)	1 (16.7%)	9 (16.4%)
Age 18 years or older, n (%)	6 (18.8%)	3 (17.6%)	0 (0%)	9 (16.4%)

* Drug exposure refers here typically to exposure to opioids, either as illegal use or a part of opioid treatment program, and benzodiazepines. Opioids, especially buprenorphine and benzodiazepines, are the main drugs used in Finland by mothers [26] ** Of those born abroad, two were born in Africa, but all others were from eastern European countries near Finland, and the study population was thus ethnically homogenous.

**Table 2:** The five most common ICD-10 three-digit additional diagnoses/diagnose groups for hospital visits/admissions, rehabilitations, and regular medications in each age category. Some patients have incomplete follow-up times in each category. All middle ear infections have been counted as otitis media. * Note that in category 0–16 years, all patients regardless of follow-up time are included, meaning that some problems have not yet emerged by the time of data collection.

	<1 Year n = 43	1–6 Years n = 45	7–12 Years n = 37	13–16 Years n = 15	0–16 Years * n = 55
Most common diagnosis, n (%)	P04 Fetus and newborn affected by noxious influences	F82 Developmental disorder of motor function	F90 Hyperkinetic disorders (ADHD)	F90 Hyperkinetic disorders (ADHD)	F90 Hyperkinetic disorders (ADHD)
	23 (53.5%)	18 (40%)	25 (67.6%)	10 (66.7%)	27 (49.1%)
Second most common diagnosis, n (%)	P07 Short gestation and low birth weight	F80 Developmental disorders of speech and language	F81 Developmental disorders of scholastic skills	F70 Mild intellectual disabilities	P04 Fetus and newborn affected by noxious influences
	15 (34.9%)	15 (33%)	12 (32.4%)	8 (53.3%)	23 (41.8%)
Third most common diagnosis, n (%)	R62 Lack of expected normal development	F83 Mixed developmental disorders	F70 Mild intellectual disabilities	F81 Developmental disorders of scholastic skills	F82 Developmental disorder of motor function
	12 (27.9%)	14 (31.1%)	8 (21.6%)	4 (26.7%)	18 (32.7%)
Fourth most common diagnosis, n (%)	P05 Slow fetal growth	H50 Other strabismus	F94 Disorders of social functioning	F32 Depressive episode	F81 Developmental disorders of scholastic skills
	11 (28.9%)	12 (26.7%)	5 (13.5%)	3 (20%)	16 (29.1%)
Fifth most common diagnosis, n (%)	H66/H65 Otitis media	H66/H65 Otitis media	F83 Mixed developmental disorders	F94 Disorders of social functioning	H65/H66 Otitis media
	10 (23.3%)	10 (22.2%)	5 (13.5%)	2 (13.3%)	16 (29.1%)
Physiotherapy, n (%)	29 (72.5%)	6 (13.3%)	0	0	31 (56.4%)
Speech therapy, n (%)	-	25 (55.6%)	0 (0%)	2 (13.3%)	26 (47.3%)
Occupational therapy, n (%)	-	39 (86.7%)	2 (5.4%)	1 (6.7%)	40 (72.7%)
Psychosocial therapy, n (%)	-	2 (4.4%) (Theraplay)	2 (5.4%)	1 (6.7%)	5 (9.1%)
Neuropsychologic rehabilitation n (%)	.	-	2 (5.4%)	0	2 (3.6%)
First out-of-home placement, n (%)	23 (53.5%)	17 (37.8%)	3 (8.1%)	1 (6.7%)	44 (80%) (all with any placements)
Psychostimulant medication, n (%)	-	3(6.7%)	24 (64.9%)	5 (33.3%)	27 (49.1%)
Risperidone medication, n (%)	-	1 (2.2%)	4 (10.8%)	3 (20%)	6 (10.9%)
Other psychiatric medication, n (%)	-	-	3 (8.1%)	4 (26.7%)	6 (10.9%)
Valproate for epilepsy, n (%)	0	1 (2.2%)	2 (5.4%)	0	2 (3.6%)
Growth hormone, n (%)	-	1 (2.2%)	1 (2.7%)	1 (6.7%)	3 (5.5%)
Insulin, n (%)	0	1 (2.2%)	1 (2.7%)	1 (6.7%)	1 (1.8%)

**Table 3:** Mean annual additional costs for age groups in patients with FASD in 2022 prices. (A) Full data set including those with incomplete follow-up times using observed costs divided by actual follow-up times. (B) Including only those with full follow-up through age group. Only additional costs caused by special needs are included, diagnosis-related costs include hospital inpatient and outpatient clinic costs, no primary health care costs. IQR = Interquartile range.

(A) Mean Annual Costs Full Data Set	<1 Year	1–6 Years	7–12 Years	13–16 Years
Follow-up time in years	39.2	232.0	148.5	42.5
Diagnosis-related costs, mean (median, IQR)	EUR 21,677 (8963, 22,772)	EUR 2094 (2010, 1747)	EUR 2991 (1971, 1455)	EUR 5567 (4278, 5409)
Out-of-home placement costs, mean (median, IQR)	EUR 35,552 (0, 72,952)	EUR 46,276 (58,564, 73,292)	EUR 48,914 (67,394, 75,360)	EUR 64,877 (79,029, 32,361)
Disability allowance costs, mean (median, IQR)	EUR 811 (612, 420)	EUR 1154 (1395, 856)	EUR 2402 (2831, 1456)	EUR 942 (1204, 1214)
Rehabilitation costs, mean (median, IQR)	EUR 1576 (2063, 1629)	EUR 2814 (3209, 2696)	EUR 849 (0, 0)	EUR 511 (0, 403)
Special support costs for school, mean (median, IQR)	EUR 0 (0, 0)	EUR 470 (0, 1584)	EUR 21,248 (17,504, 8144)	EUR 20,613 (17,690, 10,328)
Long-term medication costs, mean (median, IQR)	EUR 0 (0, 0)	EUR 175 (0, 0)	EUR 769 (640, 822)	EUR 1452 (850, 1285)
Total mean annual costs (median, IQR)	EUR 57,957 (68,344, 84,633)	EUR 51,899 (70,580, 73,767)	EUR 73,912 (81,450, 48,216)	EUR 82,394 (101,666, 88,444)
**(B)** **Mean annual costs, patients with complete follow-up**	**<1 year**	**1–6 years**	**7–12 years**	**13–16 years**
Number of patients	38	26	17	12
Diagnosis-related costs, mean (median, IQR)	EUR 21,246 (8956, 17,655)	EUR 2179 (2053, 1512)	EUR 1533 (1321, 1103)	EUR 5912 (4794, 6199)
Out-of-home placement costs, mean (median, IQR)	EUR 32,527 (0, 73,616)	43,566 EUR (63,626, 76,131)	EUR 54,971 (72,178, 76,991)	EUR 68,218 (84,022, 28,144)
Disability allowance costs, mean (median, IQR)	EUR 725 (612, 13)	EUR 981 (945, 808)	EUR 2298 (2833, 1626)	EUR 890 (1204, 1218)
Rehabilitation costs, mean (median, IQR)	EUR 1497 (2063, 2099)	EUR 1985 (1465, 2408)	EUR 392 (0, 0)	EUR 444 (0, 0)
Special support costs for school, mean (median, IQR)	EUR 0 (0, 0)	EUR 499 (0, 1600)	EUR 15,601 (16,549, 3783)	EUR 20,285 (17,647, 7454)
Long-term medication costs, mean (median, IQR)	EUR 0 (0, 0)	EUR 139 (0, 0)	EUR 758 (637, 749)	EUR 1680 (865, 1144)
Total mean annual costs (median, IQR)	EUR 55,530 (50,781, 81,269)	EUR 49,349 (71,990, 75,332)	EUR 75,283 (94,486, 74,802)	EUR 91,241 (104,484, 69,363)

## Data Availability

The data are not publicly available due to the research permit that does not allow sharing personal information.

## Appendix A. All Used Prices and Their Sources before Index Correction

For the clarity and user-friendliness, this Appendix has the sources numbered and listed as links below. The sources mentioned in the main texts are referenced also in the main reference list, but have different reference numbers there (Table A1).

**Table A1:** Prices.

Price Category	Price (EUR)	Code/Unit	Notes	Source
**HOSPITAL DIAGNOSES used for estimations**	DRG price	ICD-10 codes or category		Päijät-Häme DRG price list (1)
Concussion/mild traumatic brain injury, child	1315	S06		(1)
Seizure or headache, child	2079	G40–G44		(1)
Operation for strabismus, short stay	2660	H50		(1)
Other ophthalmological disease	2789	H51–H54, Q13–Q14		(1)
Tonsillectomy/Adenoidectomy	2596	J35–J36		(1)
Otitis media, complicated, or tympanostomy, child	2302	H65–H66		(1)
Pneumonia or pleuritis, child	3805	J09–J18		(1)
Obstructive bronchitis/bronchiolitis or acute asthma, child	2918	J20–J22, J40–J47		(1)
Other ear–nose–throat disease, child	2168	Q32		(1)
Congenital heart defect, child	6024	Q20–Q28		(1)
Operation for hernia, child	6367	K40–K46		(1)
Appendectomy, uncomplicated	4223	K35–K37		(1)
Juvenile diabetes, young patient	4465	E10		(1)
Urinary tract infection/disease, short stay, no significant operation	1085	N10, N30, N39		(1)
Testicular operation for non-malignant cause, child	4143	Q53, N44		(1)
Newborn, birthweight <1000 g	146,652	P07.0		(1)
Newborn, birthweight 1000–1499 g	57,785	P07.1		(1)
Newborn, birthweight 1500–2499 g, problems in multiple organ systems	41,760	P07.3	Several other diagnoses, long NICU stay	(1)
Newborn, birthweight 1500–2499 g, no problems in multiple organ systems	17,058	P07.3	Short NICU stay, no severe complications	(1)
Newborn, birthweight 2500 g or more, problems in multiple organ systems	21,179	long NICU stay, no P07	Severe congenital problems or long morphine treatment for neonatal abstinence syndrome	(1)
Newborn, birthweight 2500 g or more, other significant problem	7632	Short <7 days of NICU stay, no P07	For example, transient respiratory problems, need for IV glucose or IV antibiotics for an infection	(1)
Intellectual disability	2628	F70–F79		(1)
Neuropsychiatric disorder	4834	F84–F90		(1)
Other psychiatric, emotional or behavioral disorder in childhood	5261	F91–F99		(1)
Mood or anxiety disorder	3123	F30–F48		(1)
Intellectual disability	2628	F70–F79		(1)
Traumatic injury (other than concussion), child	1017	S-(except S06) and T-codes		(1)
Poisoning or toxic effect of a substance	1439	X44, Y91		(1)
Neurologic disease or disorder, without significant complications	1090	R62, Q86, F80, F81, F82, Q02		(1)
Pregnancy related complication	1274	O24		(1)
Eating disorder	8424	F50		(1)
Gastrointestinal disease, short stay without significant operation	754	A08, A09, K5		(1)
Other central nervous system disorder, uncomplicated	2250	F83		(1)
Endocrine disorder, short treatment, without significant operation	911	E34, E46, E23		(1)
Other or unspecified issue, short treatment without significant operation	722	UNSPECIFIED	Other diagnoses not listed here	(1)
Day in adolescent psychiatric ward	681	price/day		(1)
Brain MRI with anesthesia	750	MRI		(1)
Sleep–wake EEG registration	300	EEG		(1)
Molecular karyotype/chromosomal microarray	800	Mksyn		(1)
DRGHUS Esophagus atresia	5851	Q39	Operated in HUS	(2)
DRGHUS Cleft lip	6327	Q36	Operated in HUS	(2)
2. **REHABILITATIONS**	Price for a typical rehabilitation period	Price per unit and number of units		Primary care (1). Kela/SII (3, 4)
Psychiatric rehabilitation/psychotherapy	19,014		If psychotherapy	(1)
Physical therapy, in primary health care	1965	EUR 131/45 min × 15	If only between 1 and 12 months	(1)
Physical therapy, via Kela	2400	EUR 60/45 min × 40	If physical therapy, 1–6 years	(3, 4)
Speech therapy, in primary health care	6360	EUR 212/45 min × 30	If no diagnosis F80 or F83 1–6 years	(1)
Speech therapy, via Kela	9600	EUR 120/45 min × 80	If F80 or F83 or F7 1–6 years, if 7+ years, additional 4800 is added	(3, 4)
Occupational therapy, in primary health care	5640	EUR 141/45 min × 40	If no diagnosis F82 or F83	(1)
Music therapy	4600	EUR 115/45 min × 40		(1)
Neuropsychological rehabilitation	9280	EUR 116/45 min × 80		(3, 4)
Theraplay	3360	EUR 168/60 min × 20		(1)
3. **SPECIAL NEEDS SCHOOL ARRANGEMENTS**	Price	Time	Estimation and what is included	Finnish National Agency for Education: Prices and Funding (5), Statistics (6, 7, 8, 9)
Special school for severely disabled (category 1)	28,021	year	Includes taxi transfers, morning, evening and holiday day care, and structural costs associated with special school	(5)
Extra year of kindergarten as a part of extended compulsory education	9221	year	Added once to those in categories 1 and 2	(6)
Extended compulsory education for disabled (category 2)	18,584	year	EUR 28,021–9437 (basic price)	(7)
Special needs education special support in smaller class, regular school (category 3)	13,645	year	One teaching price subtracted from the price in category 2	(6, 7)
Special support inclusion/integration to mainstream classroom (category 4)	4939	year	Only teaching costs are doubled	(6, 7)
Intensified support in mainstream classroom (category 5)	2564	year	Cost for flexible teaching arrangements	(6, 7)
Extra school year (repeating a grade)	9437	year	Basic price for a schoolyear	(6)
Care for a special needs student in the afternoons after school day	1568	year	Grades 2–3 if diagnosed with F83/F81.3 or F90, and grades 2–9 if diagnosed with F70–F79, in categories 2–5 (included in the price in category 1)	(8)
Taxi transfer to school and back home	1303	year	For those diagnosed with F70–F79 and F83/F81.3 and F90 in categories 2–5 (included in the price in category 1)	(9)
4. **DISABILITY ALLOWANCE**	Price/year	Price/month		Kela/SII (10)
Disability allowance, basic	1145	95.42	A total of 0.5 year if long-term problems during infancy, after 3 years for others with developmental (F70–F84) diagnosis and medication or 1 therapy or psychiatric diagnosis	(10)
Disability allowance, middle	2671	222.60	Ages 3–16 if diabetes or developmental diagnosis (F70–F84) and at least two therapies or psychiatric care and medication or therapy	(10)
Disability allowance, highest	5071	422.58	If needs round-the-clock care for severe disability or diabetes; 0–3 years	(10)
5. **OUT-OF-HOME PLACEMENT**	Price/year (nominal prices)	Price/month (nominal prices)	Mean price per 0–17-year-old person placed out of home in the Hospital catchment area	Finnish Institute on health and welfare and Statistics of Finland (11, 12) and Kuusikko statistics (13)
Out-of-home placement 2006	47,890	3990		(11, 12)
Out-of-home placement 2007	46,670	3890		(11, 12)
Out-of-home placement 2008	56,160	4680		(11, 12)
Out-of-home placement 2009	57,940	4830		(11, 12)
Out-of-home placement 2010	58,040	4840		(11, 12)
Out-of-home placement 2011	62,260	5190		(11, 12)
Out-of-home placement 2012	64,710	5390		(11, 12)
Out-of-home placement 2013	59,090	4924		(11, 12)
Out-of-home placement 2014	60,990	5080		(11, 12)
Out-of-home placement 2015	72,090	6010		(11, 12)
Out-of-home placement 2016	78,700	6560		(11, 12)
Out-of-home placement 2017	73,900	6160		(11, 12)
Out-of-home placement 2018	74,530	6211		(11, 12)
Out-of-home placement 2019	66,900	5580		(11, 12)
Out-of-home placement 2020	72,810	6070		(11, 12)
Expensive special institutional placement, when length is known	Each year’s nominal price multiplied by 1.5	Each month’s nominal price multiplied by 1.5	Estimated as having 1.5 times the mean price, using prices from other Finnish cities	(13)
6. **MEDICATION COSTS** **(rounded to nearest full hundred)**	Price	unit	All costs including required health checks and laboratory tests, diagnostic cost not included (included in DRG price of a diagnosis)	All pharmacies have same prices, Yliopiston apteekki used as source (14), Unit costs of health and social care in Finland (15), Medication statistics (16)
ADHD medication (methylphenidate, typical dosage 40 mg)	600	year	Medication price for 12 months and health check by school nurse	(14, 15, 16)
Risperidone (typical dosage 1 mg/day)	500	year	Medication price for 12 months, safety laboratory follow-up tests, two health checks by nurse, doctor phone control	(14, 15, 16)
Other antipsychotic behavioral medications (aripipratzole, quetiapine)	200	year	Medication price for 12 months, nurse’s check and doctor phone control	(14, 15, 16)
SSRI (fluoxetine used typically in children and adolescents)	200	year	Medication price for 12 months, nurse’s check and doctor phone control	(14, 15, 16)
Valproate medication	1000	year	Doctor’s appointment once a year and nurse’s check once a year, laboratory checks occasionally after first treatment year, occasional EEG-registrations included, not including hospitalizations in case of seizures	(14, 15, 16)
Insulin	2900	year	Not including complications or hospitalizations at diagnosis or later due to complications	Price of diabetes in Finland, (17).
Growth hormone	12,000	year	Including health checks, laboratory safety follow-up tests and bone age X-rays.	(14, 15, 16)

Sources for prices as links:

1. https://paijat-sote.fi/wp-content/uploads/2021/05/palveluhinnasto_2021.pdf
2. https://www.hus.fi/sites/default/files/2020-09/HUS%20Palveluhinnasto%202020%2C%20tuote-%20ja%20suoriteperusteiset%20hinnat%20%28osat%201%20ja%202%29.pdf
3. https://raportit.kela.fi/ibi_apps/WFServlet?IBIF_ex=NIT099AL&YKIELI=E
4. https://www.kela.fi/valitut-palveluntuottajat_etelainen
5. https://www.oph.fi/sites/default/files/documents/opetus_ja_kulttuuritoimen_rahoitus_2020.pdf
6. https://vos.oph.fi/rap/kust/v20/raportit.html
7. https://vos.oph.fi/rap/kust/v20/k05r6vm.html
8. https://vos.oph.fi/rap/kust/v20/k55n6nmk.html
9. https://vos.oph.fi/cgi-bin/tiedot2.cgi?saaja=3983;tnimi=kust/v20/k05k7s.lis
10. https://www.kela.fi/etti/Alle16-vuotiaanvammaistuki.pdf?version=1664542286228
11. https://sotkanet.fi/sotkanet/en/taulukko/?indicator=szaytfbzBQA=&region=s7YsAQA=&year=sy5zsk7S0zUEAA==&gender=t&abs=f&color=f&buildVersion=3.1.1&buildTimestamp=20221109102
12. https://pxdata.stat.fi/PxWeb/pxweb/en/StatFin/StatFin__vaerak/statfin_vaerak_pxt_11re.px/
13. https://www.hel.fi/hel2/tietokeskus/julkaisut/pdf/22_06_01_Kuusikko_Lastensuojelu_2021.pdf
14. https://www.yliopistonapteekki.fi/
15. https://www.julkari.fi/bitstream/handle/10024/142882/URN_ISBN_978-952-343-493-6.pdf?sequence=1&isAllowed=y
16. https://www.julkari.fi/bitstream/handle/10024/145776/Suomen_laaketilasto_2021.pdf
17. https://sely.fi/wp-content/uploads/2018/03/el418_165.pdf

More detailed explanations for prices used (references are listed according to the source links above, all accessed on 20 January 2023).

Hospitalization costs/diagnosis-related cost: Hospitalization and hospital visit costs were estimated using available DRG prices for each main diagnosis listed in hospital clinic outpatient visit or hospital ward inpatient stay during each age group using the 2021 price list for services in the Päijät-Häme area (1). All adolescents diagnosed with F94.1 (Reactive attachment disorder), F32.9 (Depressive episode) and F50.0 (Anorexia nervosa) needed long inpatient treatment in the adolescent psychiatric unit, which is significantly more expensive than the DRG price based on adult patients. For a more accurate estimation of those diagnoses, instead of using diagnosis-based DRG price, we used the DRG price for adolescent psychiatric ward multiplied by the length of stay. In addition to diagnosis-based DRG prices, we added costs from sleep–wake EEG, brain MRI and molecular karyotyping in hospital costs depending on the age at which they were performed. For the operations performed in Helsinki university hospital (HUS), the price list for HUS (2) was used.

Rehabilitations/therapies: Because exact number of rehabilitations was not easily available, the numbers were estimated using the typical rehabilitation schemes in our hospital district. In Finland, rehabilitation for severe developmental problems (intensive medical rehabilitation, vaativa lääkinnällinen kuntoutus in Finnish) is financed via SII, and for mild or intermediate difficulties, it is arranged via either hospital or basic health care and financed by hospital district. For treatments financed by hospital district, prices were obtained from pricelist for services in Päijät-Häme area 2021 (1). For those financed via SII, prices for rehabilitation are estimated as averages for that type of rehabilitation in the hospital district area from SII Rehabilitation services arranged by Kela statistics (3) and SII Procurement decisions of the Southern Insurance District 2018 (4). Physical therapy (infant) cost is EUR 131/45 min, for those with the diagnosis of R62/45 min sessions (via hospital) (EUR 1965). For those who received physical therapy after the age of 1 year (via SII), the cost is EUR 60/45 min multiplied by 40 (EUR 2400). Speech therapy, for those with the diagnosis of F80 or F83 (via SII), costs EUR 120/45 min multiplied by 80 (two years of therapy) during preschool age (EUR 9600), and for those without the diagnosis of F80 nor F83 (via basic health care), the cost is EUR 212/45 min multiplied by 30 (EUR 6360). For those over 7 years of age, the cost is multiplied by 40 (one year of therapy) via SII (EUR 4800). Occupational therapy, for those with the diagnosis of F82 or F83 (via SII), is EUR 80/45 min multiplied by 120 (three years of therapy) during preschool age (EUR 9600), and for those without those diagnoses (via basic health care), it is EUR 141/45 min multiplied by 40 (EUR 5640). For those over 7 years of age, it is multiplied by 40 (one year of therapy) via SII (EUR 3200). Music therapy costs EUR 115/45 min multiplied by 40, same for those over 7 years of age (EUR 4600) (1). Theraplay costs EUR 168/60 min multiplied by 20 (EUR 3360) (1). Neuropsychological rehabilitation costs EUR 116/45 min multiplied by 80 (EUR 9280) (via SII). Psychotherapy DRG total price is EUR 19 014 (1).

Special needs school arrangements: School support prices are calculated as difference between regular student and special needs student in different support levels. Prices are based on statistics and cost reports of Finnish National Agency for Education (https://www.oph.fi/en/statistics-and-publications, accessed on 20 January 2023) and Opetustoimen kustannusraportit vuodelta 2020, Päijät-Häme (6–9). Because in official price lists only three levels (regular price, price for those with extended compulsory education and price for those with severe disability) of annual prices according to support level are listed, other levels were estimated. There are also annual prices for private and state-owned specialty schools available, and those were used as basis for estimating specialty school prices.

The explanation of funding system in Finnish school can be found in Finnish in Opetus- ja kulttuuritoimen rahoitus 2020 (5).

Most costs in special education occur because of personnel costs. Smaller groups equal higher costs, and each assisting person needed in classroom causes extra costs. In special needs education classroom in a regular school there are usually 8–10 pupils, 1 teacher and 1–2 teacher’s assistants. For those with extended compulsory education for disabled and those in specialized schools, there are typically 5–8 pupils in a classroom, 1 teacher and 1–4 teacher’s assistants. Prices for the main city (Lahti) in the area were used (9).

In special schools for the disabled (category 1), the price for those with severe disability of EUR 28021/year was used without subtracting the basic price, because additional infrastructural costs are associated with special school, and high teaching costs are caused by the low pupil/adult ratio. Because all students in special schools are entitled to taxi transfers and care before and after school, those prices are included in the total cost. This estimate is significantly lower than the average price per pupil in the private or state-owned special schools for disabled, which is EUR 61,349 (Valteri EUR 106,218, Sylvia-koti EUR 36,637, State-owned reform schools EUR 41,191), but because those operate also as boarding schools and have some boarding pupils, including them would overestimate real costs.

Extended compulsory education for the disabled (category 2) costs EUR 18,584/year, which is the same price as that of special schools for the disabled, because, similarly, there are very small classes with low pupil/adult ratio, individual syllabus and education plan, but the price of regular schoolyear (EUR 9437/year) was subtracted, because regular school infrastructure is used.

Special needs education in a small class (category 3) costs EUR 13,645/year. The price is estimated by taking the mean price for category 2 and subtracting form it one teaching price per pupil because of slightly higher pupil/adult ratio (EUR 4952).

Special support and inclusion/integration to mainstream classroom (category 4) costs EUR 4952/year. For those who are integrated into mainstream classroom, estimated extra costs occur, for example, for extra lessons (typically once a week during a schoolyear), extra materials, simplified books, electric books, computer or tablet computer, special teaching materials, extra hours of teacher’s time needed for arranging and having network meetings with parents, therapists, school health personnel and making the obligatory paperwork 5 h/year, extra support by school health personnel (school psychologist, counsellor, and nurse), separate lessons in small group with a special teacher. Estimated sum of EUR 5000/year is very close to doubling the teaching costs (EUR 4952/year) per student, which is why it was used for the cost.

Intensified support at school in mainstream classroom or flexible teaching at middle school (category 5) costs EUR 2564/year, which includes extra lessons and extra hours of teacher’s time needed for arranging and having network meetings with parents and school health personnel when needed.

One additional schoolyear costs EUR 9437/year, the price of regular schoolyear in the hospital district. Possible support levels are added.

Extra year in kindergarten for those with extended compulsory education for disabled meaning two years of special needs kindergarten before school in special needs education class costs EUR 9221 per person.

Taxi transfer to and from school (included in the price in special schools) costs EUR 1303/year per person for grades 1–9 for those diagnosed with intellectual disability and for grades 1–3 for those diagnosed with global learning disabilities and ADHD.

Morning and afternoon activities for special needs students before and after school day between 07 and 17 o’clock (included in the price in special schools) costs EUR 1568/year for those with intellectual disability for grades 2–9 (8 years). For those diagnosed with global learning disabilities or ADHD, the care is calculated for grades 2–3 (2 years). Afternoon care for first graders is a general service available for all and thus not calculated as an extra cost.

Disability allowance: For calculating disability allowance, we used the SII prices for 2020 and the criteria listed in 15 December 2021 (10): Basic level is EUR 95.42/month (EUR 1145/year). For six months of that level in infancy, physical therapy or a long treatment in NICU is required (cost more than EUR 20,000) or >2 hospitalizations. It is also the level that concerns those aged over three years with only one type of rehabilitation or just intellectual disability is observed or psychiatric care is needed (one type of significant problem or support is required). Medium price level is EUR 22,260/month (EUR 2671/year). For that level two therapies are required between 3 and 6 years, or one therapy and intellectual disability or diabetes or psychiatric care need treatment (at least two types of problems or supports are required). The highest cost is EUR 42,258/month (EUR 5071/year), which is for patients needing round-the-clock care, for most severe disabilities and for diabetes, at 0–3 years (no such patients were among this study population).

Out-of-home placements: Out-of-home placement costs per 0–17-year-old person in Päijät-Häme area were on average EUR 65,800/year or EUR 5483 per month in 2009–2020 (11,12). For known exact length periods of institutional placement, that was multiplied by 1.5, estimated using prices for six big cities in Finland (13).

Medication: Medication cost (rounded to nearest full hundred) was calculated using the price for typical dosage in pharmacies (same price in all pharmacies in Finland for all prescription medications available in any Finnish pharmacy webpage (14)) and typical possible health checks and laboratory test prices were included according a general price list from 2017 (the most recent publicly available data set). Costs of health and social care in Finland (15) are unified according to Medication statistics in Finland (16), except for insulin, in which a research-based price of EUR 2900/year was available (17), including regular checks, medication and equipment, but not including complications or hospitalizations at diagnosis or later due to complications. For human growth hormone (somatropin), the price of EUR 12,000/year included medication, health checks, blood tests and bone age X-rays.

## References

[1] Hoyme H.E., May P.A., Kalberg W.O., Kodituwakku P., Gossage J.P., Trujillo P.M., Robinson L.K. (2005). A practical clinical approach to diagnosis of fetal alcohol spectrum disorders: Clarification of the 1996 Institute of Medicine criteria. Pediatrics.

[2] Koponen A., Kalland M., Autti-Rämö I. (2009). Caregiving environment and socio-emotional development of foster-placed FASD-children. Child. Youth Serv. Rev..

[3] Hoyme H.E., Kalberg W.O., Elliott A.J., Blankenship J., Buckley D., Marais A.S., May P.A. (2016). Updated clinical guidelines for diagnosing fetal alcohol spectrum disorders. Pediatrics.

[4] May P.A., Hasken J.M., Baete A., Russo J., Elliott A.J., Kalberg W.O., Hoyme H.E. (2020). Fetal alcohol spectrum disorders in a Midwestern city: Child characteristics, maternal risk traits, and prevalence. Alcohol. Clin. Exp. Res..

[5] Hemingway S.J.A., Bledsoe J.M., Brooks A., Davies J.K., Jirikowic T., Olson E., Thorne J.C. (2019). Comparison of the 4-digit code, Canadian 2015, Australian 2016 and Hoyme 2016 fetal alcohol spectrum disorder diagnostic guidelines. Adv. Pediatr. Res..

[6] Autti-Rämö I., Fagerlund A., Ervalahti N., Loimu L., Korkman M., Hoyme H.E. (2006). Fetal alcohol spectrum disorders in Finland: Clinical delineation of 77 older children and adolescents. Am. J. Med. Genet. Part A.

[7] Lange S., Probst C., Gmel G., Rehm J., Burd L., Popova S. (2017). Global Prevalence of Fetal Alcohol Spectrum Disorder Among Children and Youth: A Systematic Review and Meta-analysis. JAMA Pediatr..

[8] Popova S., Lange S., Shield K., Mihic A., Chudley A.E., Mukherjee R.A., Rehm J. (2016). Comorbidity of fetal alcohol spectrum disorder: A systematic review and meta-analysis. Lancet.

[9] Streissguth A.P., Bookstein F.L., Barr H.M., Sampson P.D., O’malley K., Young J.K. (2004). Risk factors for adverse life outcomes in fetal alcohol syndrome and fetal alcohol effects. J. Dev. Behav. Pediatr..

[10] McLachlan K., Flannigan K., Temple V., Unsworth K., Cook J.L. (2020). Difficulties in daily living experienced by adolescents, transition-aged youth, and adults with fetal alcohol spectrum disorder. Alcohol. Clin. Exp. Res..

[11] Popova S., Stade B., Bekmuradov D., Lange S., Rehm J. (2011). What do we know about the economic impact of fetal alcohol spectrum disorder? A systematic literature review. Alcohol Alcohol..

[12] Ericson L., Magnusson L., Hovstadius B. (2016). Societal costs of fetal alcohol syndrome in Sweden. Eur. J. Health Econ..

[13] Frappier J., Tremblay G., Charny M., Cloutier L.M. (2015). Costing bias in economic evaluations. J. Med. Econ..

[14] Official Statistics of Finland (OSF) (2023). Price Index of Public Expenditure, Local Government Finances by Function Area, Old Annual Data, 1975–2022.

[15] Official Statistics of Finland (OSF) (2023). Consumer Price Indices, Overall Index, Yearly Data, 1972–2022.

[16] R Core Team (2020). R: A Language and Environment for Statistical Computing.

[17] (2021). Päijät-Hämeen Hyvinvointiyhtymä: Palveluhinnasto 2021; Price List for Services. https://paijat-sote.fi/wp-content/uploads/2021/05/palveluhinnasto_2021.pdf.

[18] Sotkanet (2022). Sotkanet.fi Statistics and Indicator Bank. The Finnish Institute for Health and Welfare 2005–2022. Institutional and Family Care in Child Welfare, Total Operating Expenditure, Placements Outside the Home for Those Aged 0–17, as % of Total Population of Same Age. Päijät-Häme Hospital Distict. https://sotkanet.fi/sotkanet/en/taulukko/?indicator=szaytfbzBQA=&region=s7YsAQA=&year=sy5zsk7S0zUEAA==&gender=t&abs=f&color=f&buildVersion=3.1.1&buildTimestamp=202211091024.

[19] Official Statistics of Finland (OSF) (2022). Population Structure.

[20] Kuusikko-Työryhmä (2022). Kuuden Suurimman Kaupungin Lastensuojelun Palvelut ja Kustannukset Vuonna 2021. [Child Care Services and Costs in the Six Largest Cities in 2021].

[21] Kela (2021). Alle 16-Vuotiaan Vammaistuki 15.12.2021 [Disability Allowance for Those under 16 Years]. https://www.kela.fi/etti/Alle16-vuotiaanvammaistuki.pdf?version=1664542286228.

[22] Kela (2021). Valitut Palveluntuottajat Vakuutuspiirien Järjestämissä Vaativan Lääkinnällisen Kuntoutuksen Terapiahankinnoissa 2018/Eteläinen Vakuutuspiiri. [SII’s Procurement Decisions of the Southern INSURANCE District Year 2018]. https://www.kela.fi/valitut-palveluntuottajat_etelainen.

[23] Kela (2022). Rehabilitation Services Arranged by Kela: Number of Clients and Total Expenditure. https://raportit.kela.fi/ibi_apps/WFServlet?IBIF_ex=NIT099AL&YKIELI=E.

[24] Opetushallitus (2020). Opetus-ja Kulttuuritoimen Rahoitus-Yksikköhintojen ja Rahoituksen Määräytyminen Vuonna 2020. [Finnish National Agency for Education. Determination of Teaching and Cultural Activity Funding Unit Prices and Funding in 2020]. Oppaat ja Käsikirjat 2020:5. https://www.oph.fi/sites/default/files/documents/opetus_ja_kulttuuritoimen_rahoitus_2020.pdf.

[25] Opetushallitus (2020). Kustannustilastot 2020. [Finnish National Agency for Education.Statistics of Expenses 2020]. https://vos.oph.fi/rap/kust/v20/raportit.html.

[26] Basnet S., Onyeka I.N., Tiihonen J., Föhr J., Kauhanen J. (2015). Characteristics of drug-abusing females with and without children seeking treatment in Helsinki, Finland. Scand. J. Public Health.

[27] Reid N., Dawe S., Shelton D., Harnett P., Warner J., Armstrong E., O’Callaghan F. (2015). Systematic review of fetal alcohol spectrum disorder interventions across the life span. Alcohol. Clin. Exp. Res..

[28] Hannigan J.H., Armant D.R. (2000). Alcohol in pregnancy and neonatal outcome. Seminars in Neonatology.

[29] Burd L., Deal E., Rios R., Adickes E., Wynne J., Klug M.G. (2007). Congenital heart defects and fetal alcohol spectrum disorders. Congenit. Heart Dis..

[30] Liu Y., Chen S., Zühlke L., Black G.C., Choy M.K., Li N., Keavney B.D. (2019). Global birth prevalence of congenital heart defects 1970–2017: Updated systematic review and meta-analysis of 260 studies. Int. J. Epidemiol..

[31] Gyllencreutz E., Aring E., Landgren V., Svensson L., Landgren M., Grönlund M.A. (2020). Ophthalmologic findings in fetal alcohol spectrum disorders–a cohort study from childhood to adulthood. Am. J. Ophthalmol..

[32] Hashemi H., Pakzad R., Heydarian S., Yekta A., Aghamirsalim M., Shokrollahzadeh F., Khabazkhoob M. (2019). Global and regional prevalence of strabismus: A comprehensive systematic review and meta-analysis. Strabismus.

[33] Strömland K., Pinazo-Durán M.D. (2002). Ophthalmic involvement in the fetal alcohol syndrome: Clinical and animal model studies. Alcohol Alcohol..

[34] Aring E., Gyllencreutz E., Landgren V., Svensson L., Landgren M., Grönlund M.A. (2021). The FASD Eye Code: A complementary diagnostic tool in fetal alcohol spectrum disorders. BMJ Open Ophthalmol..

[35] Lehikoinen A., Sorri I., Voutilainen R., Heinonen S. (2021). Optical coherence tomography shows decreased thickness of retinal nerve fibre layer among foetal alcohol exposed young adults in a case–control study. Acta Ophthalmol..

[36] Harrington S., Davison P.A., O’Dwyer V. (2022). School performance and undetected and untreated visual problems in schoolchildren in Ireland; a population-based cross-sectional study. Ir. Educ. Stud..

[37] Church M.W., Eldis F., Blakley B.W., Bawle E.V. (1997). Hearing, language, speech, vestibular, and dentofacial disorders in fetal alcohol syndrome. Alcohol. Clin. Exp. Res..

[38] Sarkola T., Gissler M., Kahila H., Autti-Rämö I., Halmesmäki E. (2011). Early healthcare utilization and welfare interventions among children of mothers with alcohol and substance abuse: A retrospective cohort study. Acta Paediatr..

[39] Rosenfeld R.M., Shin J.J., Schwartz S.R., Coggins R., Gagnon L., Hackell J.M., Corrigan M.D. (2016). Clinical practice guideline: Otitis media with effusion (update). Otolaryngol.-Head Neck Surg..

[40] Long X., Little G., Beaulieu C., Lebel C. (2018). Sensorimotor network alterations in children and youth with prenatal alcohol exposure. Hum. Brain Mapp..

[41] Jirikowic T., Olson H.C., Kartin D. (2008). Sensory processing, school performance, and adaptive behavior of young school-age children with fetal alcohol spectrum disorders. Phys. Occup. Ther. Pediatr..

[42] Robinson G.C., Conry J.L., Conry R.F. (1987). Clinical profile and prevalence of fetal alcohol syndrome in an isolated community in British Columbia. CMAJ Can. Med. Assoc. J..

[43] Streissguth A.P. (1994). A long-term perspective of FAS. Alcohol Health Res. World.

[44] Mukherjee R.A.S., Cook P.A., Norgate S.H., Price A.D. (2019). Neurodevelopmental outcomes in individuals with fetal alcohol spectrum disorder (FASD) with and without exposure to neglect: Clinical cohort data from a national FASD diagnostic clinic. Alcohol.

[45] Autti-Rämö I., Autti T., Korkman M., Kettunen S., Salonen O., Valanne L. (2002). MRI findings in children with school problems who had been exposed prenatally to alcohol. Dev. Med. Child Neurol..

[46] Sullivan E.V., Moore E.M., Lane B., Pohl K.M., Riley E.P., Pfefferbaum A. (2020). Graded cerebellar lobular volume deficits in adolescents and young adults with fetal alcohol spectrum disorders (FASD). Cereb. Cortex.

[47] Popova S., Lange S., Burd L., Rehm J. (2015). Cost attributable to fetal alcohol spectrum disorder in the Canadian correctional system. Int. J. Law Psychiatry.

[48] OECD/European Observatory on Health Systems and Policies (2021). Finland: Country Health Profile 2021, State of Health in the EU.

[49] Varabyova Y., Müller J.M. (2016). The efficiency of health care production in OECD countries: A systematic review and meta-analysis of cross-country comparisons. Health Policy.

